# Small Bowel Evisceration after Spontaneous Vaginal Cuff Rupture

**DOI:** 10.7759/cureus.5535

**Published:** 2019-08-30

**Authors:** Brian C McMaster, Caroline Molins

**Affiliations:** 1 Emergency Medicine, AdventHealth East Orlando, Orlando, USA

**Keywords:** small bowel evisceration, vaginal cuff rupture

## Abstract

Small bowel evisceration after vaginal cuff rupture is a seldom seen surgical emergency. We report on a case of rupture seen in the emergency department in a patient eight weeks post hysterectomy. She presented to the emergency department by ambulance in the early morning hours with the complaint of acute severe abdominal pain along with nausea and vomiting. Diagnosis of this condition was confirmed after a thorough physical exam. Following manual reduction in the emergency department and immediate transfer for surgical repair helped the patient avoid the morbidity and mortality associated with this complication. This allowed her to be discharged home later the same day.

## Introduction

Small bowel evisceration after vaginal cuff rupture is a rare complication following hysterectomy. Historically, it is often seen as an early surgical complication after hysterectomy with increased mortality in patients following cuff rupture. We report a subacute case of post-operative vaginal cuff rupture in a patient presented to the emergency department with acute onset of severe abdominal pain.

## Case presentation

A 45-year-old female presented to the emergency department in the early morning hours with the complaint of acute severe abdominal pain with nausea and vomiting. The patient reported consuming a few glasses of wine prior to going to sleep earlier that night and awoke in the middle of the night with upper abdominal pain. She reported going to her bathroom and experienced retching without vomiting. The patient had previously experienced inability to vomit following gastric sleeve placement approximately 10 years prior. Following the “retching” episodes where she was unable to vomit, she continued to have cramping abdominal pain. She noted that with the continued abdominal pain she also experienced what she thought was an episode of urinary incontinence. Concerned, the patient sat on her toilet and felt a protrusion from her vagina which she presumed to be her bladder.

The patient’s past medical history was significant for an abnormal cervical pap smear leading to a total laparoscopic hysterectomy, performed eight weeks prior to this emergency department presentation. Her obstetric history was significant for G4P2022. On review of systems, the patient reported a minor amount of vaginal spotting occurring in the few days following the surgery that resolved without intervention. The patient followed up at four weeks with the operative surgeon and was found to be healing appropriately without any noted complications.

The patient was transported by ambulance to the emergency department; she appeared uncomfortable and was experiencing a significant amount of pain. Ondansetron 4 mg and Fentanyl 250 mcg were given intravenously by paramedics en route to the hospital.

The physical examination, specifically pelvic exam, was remarkable for a protrusion of small bowel loops from the vagina. Active peristalsis of the bowel was visible on external exam. No areas of ischemic bowel were noted (Video 1).

**Video 1:** External Exam

After urgent consultation with gynecology, it was recommended the bowel be immediately reduced and the patient prepared for emergent surgical repair. Subsequently, the bowel was covered with warm, sterile saline moistened gauze, and the bowel was reduced with gentle manual pressure back into the abdominal cavity after administration of intramuscular Midazolam for procedural sedation. Based on this, the bowel was covered in warm sterile saline moistened gauze.

After successful reduction, the patient was emergently transferred to a facility with the capability to surgically repair her vaginal cuff. She was transported in reverse Trendelenburg by ambulance and asked not to perform any Valsalva maneuvers.

The operative report revealed that there was an open vaginal cuff with loops of small bowel in the upper vagina. Purulent exudative tissue was found along the margins of the previous vaginal cuff suture lines. No serosal tears were noted along the inspected areas of small bowel. Transvaginal approach was used to repair the vaginal cuff.

## Discussion

Vaginal cuff dehiscence with bowel evisceration is a rare but emergent indication for immediate surgical intervention and repair. First report dates back to 1864, and approximately 100 reports exist of this complication since that time. Rates are hard to determine due to the rarity, but literature shows a rate of between 0.032 and 1.2% of hysterectomies. Mortality increases with small bowel evisceration [1].

Pap smears are screening exams used to test for precancerous and cancerous cells of the cervix. Cervical intraepithelial neoplasia (CIN) is the term given to precancerous cells abnormally growing on the cervix following an infection of human papillomavirus. CIN is graded on a scale between 1 and 3, with 3 being the most dysplastic cells along with undifferentiated neoplastic cells. Indications for hysterectomy following identification of cervical neoplasia are multifactorial and have a wide distribution based on shared decision-making between patients and their surgeon.

Treatment of CIN varies, though higher grades of CIN require excision and ablation of the cells. Incomplete treatments that have left remaining neoplastic or dysplastic cells may require hysterectomy.

Incidence of vaginal cuff dehiscence following hysterectomy varies with approach. Studies have shown that following the increased use of minimally invasive and robotic surgeries (compared to older surgical methods), there has been a corresponding increase in rates of dehiscence. Case reports have shown occurrence of rupture ranging three days to 30 years following surgery [2].

According to a recent report by American Journal of Gynecology, hysterectomy is the second most common surgical procedure performed on women, with cesarean sections being the most commonly performed procedure [3]. Hysterectomies most often are performed for fibroids, abnormal uterine bleeding, and endometriosis. Overall the number of women undergoing this procedure from 2010 to 2013 has fallen [3].

Common events triggering dehiscence have been identified, and include sexual intercourse, defecation, increased abdominal pressure with Valsalva maneuvers or vomiting has also been a precipitating factor. Spontaneous rupture is also common [3]. No association has been noted following literature review of patients who have undergone both bariatric surgery and hysterectomy.

Dehiscence varies with surgical approach. Total transvaginal hysterectomies have less cuff dehiscence complications when compared with other less invasive approaches such as robotic assisted hysterectomy. Repair of vaginal cuff dehiscence requires emergent gynecological surgical intervention to repair cuff and to prevent ischemic changes to eviscerated bowel [4].

Initial management must include antibiotic coverage and protection of the protruding small bowel from ischemia and perforation. Warm soaked gauze should be placed on top of protruding bowel contents. Emergency stabilization with attention to prevent patient discomfort is required to keep patient calm and comfortable for transport to definitive surgical care.

Vaginal cuff dehiscence is a rare and serious post surgical complication following hysterectomy. A thorough history must be taken in patients present with acute onset of abdominal pain associated with a rush of fluid, vaginal bleeding, and acute onset of abdominal pain.

Upon rupture of the vaginal cuff, vaginal flora can easily be transferred to the abdominal cavity leading to serious complications. Abdominal peritonitis along with septicemia is possible complication following rupture.

## Conclusions

The differential diagnosis of acute onset abdominal pain is wide. A detailed history, along with thorough past surgical history that is significant for recent hysterectomy, should raise concerns for vaginal cuff dehiscence if patients present to the emergency department with sudden onset pain and rush of fluids. Prompt surgical consultation along with precautions for prevention of bowel ischemia should be emergently performed.
